# Associations Between Respiratory Muscle Strength and Maximal Oxygen Uptake Parameters in Endurance Athletes

**DOI:** 10.3390/jfmk11030274

**Published:** 2026-07-16

**Authors:** Banu Kabak, Gökhan Deliceoğlu

**Affiliations:** 1 Performance Laboratory, Department of Athlete Health Service Quality Standards, Ministry of Youth and Sports, Ankara 06080, Türkiye; 2 Department of Coaching, Faculty of Sports Sciences, Gazi University, Ankara 06500, Türkiye; gokhandeliceoglu@gazi.edu.tr

**Keywords:** endurance athlete, maximum oxygen uptake, inspiratory pressure, expiratory pressure

## Abstract

**Objectives:** The aim of this study was to investigate the association between the effect of respiratory muscle strength parameters obtained from endurance athletes and their aerobic capacity levels. **Methods:** A total of 70 endurance athletes (23 women and 47 men) voluntarily participated in the study. Respiratory muscle strength was measured using a digital spirometer. Maximum VO2 was evaluated using the cardiopulmonary exercise test system (Cosmed K5). **Results:** As a result of the study, it was determined that in female endurance athletes, Maximum Inspiratory Pressure (MIP) and Maximum Expiratory Pressure (MEP) values were related to the End-Tidal Carbon Dioxide Pressure (PETCO2) value at maximum load. In male endurance athletes, it was determined that MEP values were related to PETCO2 values at maximum load, End-Tidal Oxygen Pressure (PETO2) values at maximum load, MaxVO2 values, VO2 values at RCP, and VO2 values at Ventilatory Threshold (VT). In addition, it was determined that in male endurance athletes, the MIP value was related to the VCO2 value at Ventilatory Threshold (RCP) and the VTidal value at maximum load. The other sub-parameters of maximum oxygen consumption (VO2) examined were not found to be related to respiratory muscle strength. **Conclusions:** The present findings indicate that respiratory muscle strength may be associated with selected cardiopulmonary exercise variables in endurance athletes. However, because this was a cross-sectional study, no causal inference can be made. Prospective and intervention studies are required to clarify these associations.

## 1. Introduction

The respiratory system of healthy young individuals is generally not considered a significant limiting factor for high-intensity endurance exercise [1]. The reason for this is that the healthy lung system capacity in most people is sufficient to meet the demands for ventilation and gas exchange even during strenuous endurance exercise. The majority of untrained individuals, and even well-trained individuals, are characterized by a small increase in alveolar and arterial O_2_ difference from rest to VO2 Max of only two to three units. This small change indicates a largely uncompromised and sufficient O_2_ diffusion rate across the alveolar–capillary membrane [2].

In general, the respiratory system in healthy young individuals can be considered adequately equipped to meet the pulmonary gas exchange demands associated with even high-intensity endurance exercise. In some well-trained endurance athletes, the metabolic demand associated with high-intensity exercise may necessitate excessive ventilation and pulmonary gas exchange.

This situation may reach or exceed the functional capacity of the respiratory systems and eventually compromise arterial oxygenation and limb O_2_ transport [3].

Arterial desaturation during exercise may also occur due to an inadequate hyperventilatory response secondary to reduced chemoreceptor sensitivity (e.g., poor response to circulating chemical stimuli such as protons, catecholamines, adenosine, or potassium) [4].

In addition, mechanical restrictions presented by the airways may contribute to this condition [5]. Inadequate respiratory responses during exercise have been shown to reduce PaO_2_, negatively affecting arterial blood gas status and SaO_2_ [6]. The respiratory response during strenuous exercise, which is often accompanied and impaired by expiratory flow restrictions and dynamic hyperinflation [7], requires significant increases in both inspiratory and expiratory muscle work and can often lead to respiratory muscle fatigue [8].

However, strenuous contractions and the resulting accumulation of metabolites in the inspiratory and expiratory muscles activate unmyelinated group IV phrenic afferents [9], which reflexively increase sympathetic vasoconstrictor activity [10] and vasoconstriction in the vascular system of the exercising limb.

The result is a decrease in QL and an increase in blood flow to the respiratory muscles, suggesting a relationship that involves a struggle for limited cardiac output [11]. These effects do not occur during exercise at intensities below ~80% VO2max [12]. Fatigue-induced metabolite accumulation in respiratory muscles activates group III/IV phrenic afferents, which reflexively cause increased sympathetic efferent outflow and vasoconstriction in the extremities. This sequence facilitates locomotor muscle fatigue and limits endurance exercise performance [13].

In light of the existing literature, the aim of this study was to examine the associations between respiratory muscle strength and aerobic capacity parameters in endurance athletes. Specifically, we sought to determine whether inspiratory and expiratory muscle strength were associated with maximal oxygen uptake and selected cardiopulmonary exercise test variables. Based on previous evidence, we hypothesized that greater inspiratory and expiratory muscle strength would be positively associated with maximal oxygen uptake and related physiological parameters. However, no specific sex-based directional differences were hypothesized a priori.

## 2. Method

### 2.1. Study Design and Participant Selection

Seventy endurance athletes, 23 female and 47 male, participated voluntarily in the study. All participants were competitive endurance athletes who were actively training and competing in their respective sport disciplines at the national or regional level during the data collection period.

Ethical approval for the study was obtained from the Gazi University Ethics Committee (approval code: 1133; approval date: 13 October 2023). The study was completed in accordance with the guidelines of the Declaration of Helsinki. All participants signed an informed consent form. Inclusion criteria for the study included the following: not smoking, not having any respiratory diseases such as asthma, pulmonary tuberculosis, emphysema, and chronic bronchitis. Athletes using any medication were not included in the study. Athletes who met the study criteria were evaluated on the same day. Demographic information of the athletes (age, years of sports, number of training days per week, number of hours of training per day) was recorded. The participants included in the present study were independent of those examined in our previous publication [14] and there was no overlap between the study samples. Information about the athletes is shown in Table 1 and Table 2.

**Figure 1:**
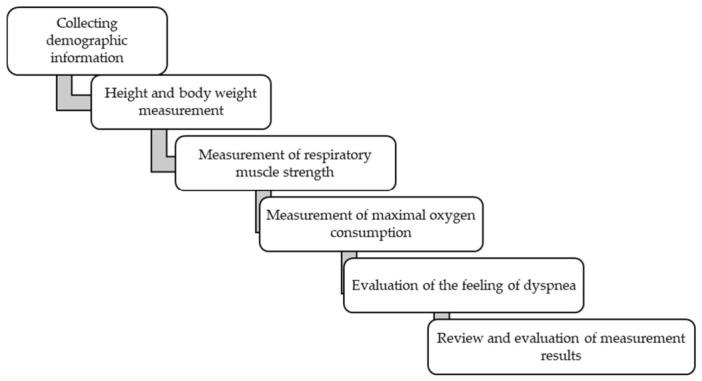
Research design workflow diagram.

Respiratory muscle strength of the athletes was evaluated with a digital spirometer (Pony FX Cosmed, Albano Laziale, Italy). Maximum oxygen uptake (MaxVO2) was assessed using the cardiopulmonary exercise testing system (Cosmed K5, Albano Laziale, Italy, Serial No: 2019030706). A Seca brand stadiometer with a precision of ±1 mm was used to measure the athletes’ heights, and a Seca (761) brand mechanical scale was used to determine the participants’ body mass. A motorized treadmill (H/p/cosmos/pulsar, Steckborn, Switzerland) was used for exercise tests with increasing workload (The steps for data collection are shown in Figure 1).

### 2.2. Height and Body Weight Measurement

A Seca brand stadiometer (Hamburg, Germany) with a precision of ±1 mm was used to measure the height of the athletes. During the measurement, the athletes were asked to step onto the device without shoes, stand in an anatomical posture, touching the overhead table with their heads at the vertex point in the frontal plane. Measurement results were recorded in cm. To determine the body weight of the athletes, a Seca (761) brand mechanical scale was used. During the measurement, the athletes were asked to step onto the device with bare feet and stand still. Data were recorded in kg.

### 2.3. Assessment of Respiratory Muscle Strength

Athletes were informed before the tests, and the tests were performed in a comfortable sitting position. During the tests, the athlete’s nose was closed with a clip, and he was asked to cover the mouthpiece with his lips, leaving no gaps at the corners of his mouth to prevent air from escaping from the spirometer mouthpiece. Tests were performed by performing respiratory maneuvers through the spirometer mouthpiece. Before the tests, a few trial tests were conducted to understand the application and adapt to the device. Participants were allowed to rest for 1 min between each trial and were asked to repeat the protocol 5 times [15].

All respiratory muscle strength assessments were performed in a standardized seated position with participants wearing a nose clip. For the MIP assessment, measurements were obtained from residual volume following a maximal expiration. For the MEP assessment, measurements were obtained from total lung capacity following a maximal inspiration. Participants performed five maneuvers for each test with one minute of recovery between attempts. The highest value obtained from technically acceptable and reproducible efforts was used for statistical analysis.

The best measurement score was used in statistical analyses.

Maximum inspiratory pressure (MIP) and maximum expiratory pressure (MEP) tests were performed to evaluate the strength of the respiratory muscles. For the MIP test, the athlete must first completely empty the air from their lungs and then take a full, deep, fast, and forceful breath. For the MEP test, the athlete was asked to first fill her/his lungs completely with air and then exhale quickly and forcefully. Each test was repeated 5 times with a rest between repetitions. Data were recorded. The best results of all tests were taken and analyzed.

### 2.4. Assessment of Aerobic Capacity (MaxVO2)

Maximum oxygen uptake (VO2 Max) was assessed using a portable cardiopulmonary exercise testing system (Cosmed K5, Albano Laziale, Italy, Serial No: 2019030706), known for its precision in automatic gas analysis of expiratory air. The calibration accuracy of the device with a known gas mixture (5.0% CO_2_ and 16.0% O_2_) was ensured. To reduce environmental influences on performance, laboratory conditions were tightly controlled with temperature maintained between 18 and 23 °C and relative humidity kept below 70% [16]. The athlete’s running was monitored in accordance with the test completion criteria used in the treadmill protocols used for aerobic capacity and power measurements. To determine the MaxVO2 parameter, after a 2 min warm-up at a speed of 6 km/h on the treadmill, they were asked to run in a protocol where the speed of the treadmill increased by 1 km/h every 90 s and the incline increased by 0.5% with this speed increase [14]. The athlete’s perceived fatigue level on the original Borg scale is 17 or above, and the athlete reports being tired. Despite increased workload, oxygen consumption no longer increases. The ratio of carbon dioxide production to oxygen consumption (RQ) reaches 1.15 or higher, heart rate is 85% or more of the maximal heart rate, and at least three of the following criteria are observed simultaneously, such as no increase in heart rate despite increased workload.

The test was concluded by accepting this as an indication that maximal oxygen utilization capacity had been reached. The average of the aerobic capacity values obtained in the last 30 s with an automatic portable gas analysis program was taken, and their ratio to the athletes’ body weights was calculated [17].

The incremental treadmill protocol used in the present study was selected because it has previously been validated for maximal cardiopulmonary exercise testing in trained endurance athletes and provides a reliable assessment of VO2max, Ventilatory Threshold (VT), and Respiratory Compensation Point (RCP). Similar protocols have been widely used in endurance performance research.

In gas analyses, minute ventilation (VE), oxygen volume per minute (VO2), and carbon dioxide volume produced per minute (VCO2) were directly measured, and the last 30 s of O_2_ use were recorded as Max VO2. The V-Slope method was used to determine the Ventilatory Threshold (VT) and Respiratory Compensation Point (RCP) [18]. After plotting the VO2 curve corresponding to VCO2 for determining VT and the VCO2 curve corresponding to VE for determining RCP, the intersection point of the two regression lines was determined by performing linear regression analysis. VO2 (mL/kg/min) value, running speed (km/h), heart rate, and running time corresponding to VT and RCP points were determined. Additionally, CO_2_ and VCO2 values at the respiratory threshold, Respiratory Compensation Point, and VE, VTidal, PETCO2, PETO2 values, and VE/VCO2, VE/VO2 ratios at maximum load were recorded.

### 2.5. Evaluation of the Feeling of Dyspnea

The Modified Borg Scale, developed by Borg [19] to measure the effort expended during physical exercise, was used to determine the feeling of dyspnea in athletes. This scale consists of ten items that define the severity of dyspnea according to its degree. The athlete was asked to rate the shortness of breath she/he felt on a scale of 0 to 10 after completing the cardiopulmonary exercise test. Data were recorded according to the athlete’s statement [19].

### 2.6. Statistical Analysis

The data collected in accordance with the hypothesis determined within the scope of the research were recorded. The minimum number of athletes to participate in the research was determined to be at least 67, calculated using the G-Power method (version 3.1.9.7). Skewness and Kurtosis values were examined to determine the distribution in the analysis part of the obtained data.

Linear regression analysis was performed to explain the relationship between the dependent variable and the independent variable and to determine how much of the variation in the dependent variable was explained by the independent variables. All statistical analyses were performed using the SPSS 26.0 software program. The study had a power of 0.80 and a margin of error of 0.05.

To account for the increased risk of Type I error arising from multiple regression analyses, the False Discovery Rate (FDR) correction proposed by Benjamini and Hochberg was applied to all reported *p*-values. Statistical significance was evaluated based on both unadjusted and FDR-adjusted *p*-values.

Prior to the regression analyses, model assumptions were evaluated. Normality of residuals was examined using Q–Q plots and the Shapiro–Wilk test. Homoscedasticity was evaluated by residual-versus-fitted plots. Multicollinearity was assessed using variance inflation factors (VIFs), while Cook’s distance values were examined to identify influential observations. No substantial violations of regression assumptions were detected.

## 3. Results

The coefficients of variation for the athletes are shown in Table 3. When the distribution of ages between groups was examined, the prevalence of females’ ages was found to be 21% on average, while the coefficient of variation for males was found to be 25%. The female group shows a more homogeneous distribution in terms of age than the male group. When the distribution of heights between groups was examined, women’s heights showed a prevalence of 3% around the mean, while the coefficient of variation for men was found to be 4%. When body weights were examined, the coefficient of variation was found to be 9% for females and 13% for males.

When the distribution of within-group variables is examined, it is seen that the female group is more homogeneous in terms of age (CV = 21.03), height (CV = 3.29), and weight (CV = 9.17) variables. When the data of the male group is examined, it is seen that while the prevalence is high according to age (CV = 25.59), it is a more homogeneous group according to the height (CV = 4.02) and weight (CV = 13.28) variables [20].

The mean inspiratory and expiratory respiratory muscle values for female and male athletes are shown in Table 4. When respiratory muscle strength values were examined, MEP and MIP values were found to be higher in female athletes than in male athletes.

Descriptive statistics of cardiopulmonary exercise test measurements in male and female endurance athletes are shown in Table 5. When the cardiopulmonary exercise test results are examined, it is seen that male athletes have higher results than female athletes. However, female athletes’ Max RQ values, VT-HR, RC-HR, and Max-HR values were found to be higher than those of male athletes.

The average respiratory frequency of female athletes was also found to be slightly lower than that of male athletes.

Table 6 shows the mean and standard deviation values of the ergospirometric parameters of the athletes.

When Table 7 is examined, it is determined that the regression model created for male and female endurance athletes in all parameters had low-level prediction rates in males and females, according to *R*^2^ values. According to the model, it was observed that MEP and MIP variables had a significant effect on PET CO2 in females, and the MEP variable in males. The MEP variable was associated with PET O2 in males, but not in females. In the study, VE at Max Load, VE/VO2 at Max Load, VT VCO2, and VE/VCO2 at Max Load were not associated with respiratory muscle strength in both males and females. VTidal at max load was associated with the MIP variable in males but not in females. In males, RCP VCO2 and VTVO2 were associated with MIP, while RCPVO2 was associated with MEP; no association was found in females for all these parameters.

## 4. Discussion

According to the literature, Bussotti et al. [21] stated in their studies on endurance athletes that the subjects with the lowest value of PETCO2 reached the lowest peak workload and VO2 during exercise [21]. They stated that athletes with the lowest PETCO2 were most likely to have the greatest exercise-induced acidosis, leading to reduced exercise capacity. Other studies have shown that endurance athletes have a well-defined ventilation pattern, spending less ventilation in dead space and thus requiring less work of the respiratory muscles [7].

Our study indicates that respiratory efficiency is related to respiratory muscle strength, especially in females. It should be noted that respiratory muscle strength, ventilatory parameters, and maximal oxygen uptake are all components of the integrated cardiorespiratory system. Therefore, the associations observed in the present study may partly reflect overall aerobic fitness, training adaptations, and cardiorespiratory conditioning rather than a direct physiological influence of respiratory muscle strength on aerobic performance. Consequently, the current findings should be interpreted as evidence of association rather than proof of a specific causal or directional mechanism. Longitudinal and intervention-based studies are needed to clarify the underlying physiological pathways.

An unexpected finding of the present study was that female athletes demonstrated higher mean MIP and MEP values than male athletes. This observation contrasts with normative reference values reported for the general population, where males typically exhibit greater respiratory muscle strength. However, the current sample consisted exclusively of highly trained endurance athletes from different sport disciplines rather than the general population. Differences in sport-specific training characteristics, respiratory muscle conditioning, and the heterogeneous distribution of sport branches between sexes may have contributed to this finding. In addition, the relatively small female sample may have amplified the influence of exceptionally high-performing individuals. Therefore, these findings should be interpreted cautiously and require confirmation in larger and more homogeneous athlete cohorts.

Females experience a higher respiratory muscle workload than males for a given absolute VE during exercise, require a higher oxygen uptake by their respiratory muscles, and have greater activation of the “extra-diaphragmatic” inspiratory muscles for relative or absolute VE during exercise, which appears to affect respiratory efficiency more than in males [22]. Studies examining the effect of respiratory muscle strength on PETO2 are quite limited; Juric et al. [23] found a significant correlation between MIP and PETO2 in their study on male basketball and handball players aged between 16 and 36 [23]. Our study differs from this study. This may be due to the difference in branches or to the fact that our athletes’ MIP values differ from the values of the athletes in Juric’s study. The blunting of the inspiratory muscle metaboreflex in healthy young women compared to men may indicate that oxygen is used more efficiently in skeletal muscles in female athletes.

An unexpected finding of the present study was that female athletes demonstrated higher mean MIP and MEP values than male athletes. This observation contrasts with normative reference values reported for the general population, where males typically exhibit greater respiratory muscle strength. A possible explanation is that the current sample consisted exclusively of trained endurance athletes rather than non-athletic individuals. Differences in sport-specific training adaptations, respiratory muscle conditioning, and the distribution of sport disciplines between sexes may have contributed to these findings. Furthermore, the relatively small number of female athletes may have amplified the influence of individual variability. Therefore, these results should be interpreted cautiously and verified in future studies involving larger and more homogeneous athlete populations.

The inverse relationship between expiratory muscles and O_2_ efficiency in men may be an indication that O_2_ utilization by expiratory respiratory muscles in male athletes is higher than in female athletes. More studies are needed to confirm these possibilities.

According to the literature, Klusiewicz [24] found no correlation between the MIP value and absolute or relative Max VO2 values in trained male athletes in his study on trained and untrained athletes, while he found a correlation between the MIP value and absolute or relative Max VO2 values in female athletes [24]. Juric et al. [23] found a significant relationship between MIP value and MaxVO2 in their studies on basketball and handball athletes [23].

On the contrary, Deliceoğlu et al. [14] did not find a significant relationship between MIP and MEP values and MaxVO2 in their study. Studies in the literature mostly describe the effect of respiratory muscle strengthening training on MaxVO2 [14].

For example, Lomax et al. [25] reported that respiratory muscle training and warming up of respiratory muscles (at 40% of maximum inspiratory muscle power) in two groups of 12 male football players improved their Yo-Yo test performance compared to the control group [25].

In their study on female rowing athletes, Volianitis et al. [26] reported that the MaxVO2 value of the athletes was higher than that of the control group after the respiratory muscle warm-up exercise performed together with the branch-specific general warm-up [26]. In contrast, Romer et al. [27] found that there was no significant change in subjects’ VO2 Max values after respiratory muscle training [27].

Females are known to have higher diaphragm, scalene, and sternocleidomastoid activation than men during graded exercise and at 85% of VO2max throughout constant load exercise and at a given VE [28].

The functional consequences of sex differences in respiratory muscle activation patterns during exercise are unclear, but increased activation of the “non-diaphragmatic” inspiratory muscles may influence the susceptibility to and magnitude of exercise-induced respiratory muscle fatigue.

When we analyze the significance of the importance weights stated in our research, it is seen that the MEP variable is significantly associated with males. The fact that the majority of expiratory muscles are also core muscles may contribute to running economy in endurance athletes.

This situation may have been associated with maximal oxygen consumption in our study, unlike other studies.

In our study, we found that the MEP variable had a significant effect on VE/VCO2 only in females. It is thought that the hyperventilation response, which is effective on the VE/VCO2 ratio, is associated with the expiratory respiratory muscle strength in females. The VE/VCO2 slope is inversely proportional to cardiac output at peak exercise [29], supporting the idea that women with morphologically smaller lungs and cardiac output mechanically support hyperventilation by increasing intrathoracic pressure with the help of expiratory muscles.

VE/VO2 ratio at maximum load can be considered as a parameter indicating ventilation efficiency. Bernardi et al. [30] found that five weeks of respiratory muscle strength training improved the VE/VO2 ratio in triathletes [30]. Bernardi’s study indicates that respiratory muscle training improves the ability to sustain exercise beyond the anaerobic threshold, with no change in either the Ventilatory Threshold or the Respiratory Compensation Point.

When we search the literature, we see that studies on the VE/VO2 ratio have mostly been conducted on patients with pulmonary hypertension. In their study, Xi et al. [31] found low VE/VO2 ratios in patients with chronic pulmonary hypertension [31]. Meyer et al. [32] report data suggesting that respiratory muscle strength is reduced in pulmonary arterial hypertension [32].

In our study, respiratory muscle strength does not affect the VE/VO2 ratio at maximum load in both genders. It is thought that the reason for this situation may be due to the endurance athletes quitting the test shortly after RCP.

As exercise intensity increases, the athlete is thought to select a combination of exercises that are assumed to maximize respiratory efficiency and minimize respiratory muscle demand [33]. Tipton et al. [34] stated that during exercise, “A carefully chosen combination of increasing frequency and tidal volume should be achieved, taking into account the need to minimize dead space ventilation” (p. 255) [34]. At the same time, this combination provides protection against excessive increases in VTidal, which would be related to excessive subatmospheric intrathoracic pressures and hence a large amount of work by the inspiratory muscles. The result is, with few exceptions, a nearly perfect and highly effective ventilatory response to exercise. Most studies have reported a plateau in VTidal before exhaustion [7]. According to our best knowledge, no study was found in the literature that directly correlated respiratory muscle strength with VTidal at maximum load. We believe that our research will contribute to the literature in this respect.

In our study, a significant relationship was found between VTidal and MIP at maximum load only in male athletes. It is thought that strong inspiratory muscles may be associated with VTidal in males. Morphological differences in the lungs and respiratory tract in females were associated with the results of our study, and it is also considered that the small number of female athletes may have been a factor.

According to the model obtained from our research, the predictive variables addressed with the VT VO2 predicted variable did not show a significant effect in female athletes, while they had a significant effect in male athletes. When the significance of the specified importance weights is examined, it is seen that the MIP variable is significantly associated with males.

According to the literature, Juric et al. [23] found a significant correlation between the VO2 value in VT and MIP in their study on male handball and basketball players [23]. This study is similar to our research. In our research, female athletes entered the respiratory threshold at an average of 84.5% of Max VO2, while male athletes entered the respiratory threshold at 82.48%. Activation of the scalenes and sternocleidomastoids has been found to be higher in women than in men at a given VE during incremental exercise [35] and during constant load exercise at 85% of VO2 Max [36]. This situation was associated with the results of our study. The consequences of gender differences in respiratory muscle activation during exercise are unclear, which may explain the gender difference found in our study.

In our study, there was no relationship between VCO2 and respiratory muscles in VT in males and females. This result may be an indication that carbon dioxide production in VT was not associated with respiratory muscle strength as a result of metabolic functions. Henke et al. [37] suggest that diaphragm fatigue during exercise is associated with increased inspiratory muscle recruitment and decreased end-expiratory lung volume [37]. During inspiration, the rib cage muscles contract while the abdominal muscles gradually relax, and the opposite happens during expiration. This mechanism prevents the rib cage from collapsing, causing the diaphragm to act as a current generator by emptying, and the abdominal volume to fall below resting levels [37,38]. The relationship between oxygen volume in the RCP and MEP can be explained by the expiratory muscles reducing the end-expiratory lung volume. This allows the diaphragm to stretch to its optimal length and allows more air to enter and perfuse the lungs. Studies are needed to explain the relationships between carbon dioxide and oxygen volumes at the Respiratory Compensation Point and respiratory muscle strength.

In our study, respiratory muscle strength at maximum load was not related to VE. Carey et al. [39] stated that high VE is achieved either by an exponential increase in respiratory frequency alone (37.5%) or by a combination of an exponential increase in respiratory frequency and a linear increase in tidal volume (62.5%) [39].

It is hypothesized that the ability to increase VE at maximal exercise in athletes may be an adaptation to training and provide the athlete with a performance advantage [40].

In our study, the sports age of female athletes was determined as ($\;\bar{X}\;$ = 9.22 ± 3.63) and the sports age of male athletes was determined as ($\;\bar{X}\;$ = 9.30 ± 5.61). This situation can be thought of as providing training adaptation.

## 5. Conclusions

For female endurance athletes, MIP and MEP variables were associated with PETCO2 at maximum load, and MEP variables affected VE/VCO2 at maximum load. For male endurance athletes, the MEP variable affects the PETCO2 variable at maximum load, the PETO2 variable at maximum load, the MaxVO2 variable, the VO2 variable in RCP, and the VO2 variable in VT. In male endurance athletes, the MIP variable affects VCO2 at RCP and VTidal at maximum load. No significant associations were observed between respiratory muscle strength and the other ergospirometric parameters examined in this study.

Research results reveal that there are relationships between maximal oxygen consumption, the most important indicator of aerobic performance, and its sub-parameters and respiratory muscles. It has been concluded that the importance of respiratory muscles should not be ignored in today’s sports competitions, where winning and losing depend on small differences.

## 6. Limitations of the Study

The main limitation of the study was that other parameters that may affect VO2 Max, such as nutrition, psychological state, and sleep, could not be examined. Other limitations include the inability to conduct a longitudinal study on the acute effects of respiratory muscle strength. In addition, the fact that the number of female athletes is less than the number of male athletes should be considered as a limitation of the study.

Another limitation of the present study is the relatively large number of regression analyses performed. Although the analyses were conducted to comprehensively explore the associations between respiratory muscle strength and cardiopulmonary exercise variables, multiple statistical comparisons may increase the risk of Type I error. Therefore, findings with marginal statistical significance should be interpreted with caution and considered exploratory until confirmed by future studies with larger and independent samples.

The relatively small number of female athletes included in the analyses (*n* = 23) should also be considered when interpreting the findings. Although the overall sample size satisfied the requirements of the a priori power analysis, the statistical power of sex-specific regression analyses may have been limited, particularly in the female subgroup. Consequently, the findings obtained for female athletes should be interpreted with caution. Future studies, including larger and more balanced samples of male and female endurance athletes, are needed to confirm the present findings and to allow the examination of potential sex-specific interactions between respiratory muscle strength and aerobic performance variables.

Although FDR correction was applied to reduce the risk of Type I error, the relatively large number of regression analyses increases the possibility that some findings may still represent chance associations. Therefore, the results should be interpreted with appropriate caution.

## Figures and Tables

**Table 1:** Number of athletes by sports branch.

Gender	Sports Branches	N
Female	Cross-Country Skiing	10
	Modern Pentathlon	11
	Triathlon	2
	Total	23
Male	Cross-Country Skiing	4
	Modern Pentathlon	9
	Biathlon	6
	Triathlon	10
	Orienteering	18
	Total	47

**Table 2:** Demographic information of the research group.

Gender	Characteristics	Min	Max	$\bar{X}$	SD
Female	Age (Years)	18	30	20.39	3.67
	Height (cm)	153	175	163.00	5.36
	Body Weight (kg)	43	63	53.35	4.89
	Sports Year (Year)	4	17	9.22	3.63
	Training Duration (Week/Day)	5	7	6.13	0.45
	Traınıng Duration (Days/Hours)	2.0	8.0	4.82	1.43
Male	Age (Years)	18	34	23.00	5.24
	Height (cm)	159	188	175.36	7.05
	Body Weight (kg)	40	85	67.45	8.96
	Sports Year (Year)	3	26	9.30	5.61
	Training Duration (Week/Day)	3	7	5.94	1.00
	Training Duration (Days/Hours)	1.0	9.0	3.63	1.83

Min: Minimum, Max: Maximum, $\bar{X}$: Average, SD: Standard deviation.

**Table 3:** Coefficients of variation of female (*n* = 23) and male (*n* = 47) endurance athletes.

Gender	Characteristics	$\bar{X}$	SD	CV (%)
Female	Age (Years)	20.39	3.67	21.03
	Height (cm)	163	5.36	3.29
	Body Weight (kg)	53.35	4.89	9.17
Male	Age (Years)	23.00	5.24	25.59
	Height (cm)	175.36	7.05	4.02
	Body Weight (kg)	67.45	8.96	13.28

$\bar{X}$: mean, SD: Standard Deviation, CV: Coefficient of Variation.

**Table 4:** Descriptive statistics of respiratory muscle test measurements of male and female endurance athletes.

Characteristics	Female				Male			
	Min.	Max.	$\bar{X}$	SD	Min.	Max.	$\bar{X}$	SD
MEP (cmH_2_O)	85.00	222.00	160.65	41.37	68	229.00	121.94	36.78
MIP (cmH_2_O)	67.00	168.00	123.57	26.44	68	206.00	100.85	30.75

MEP: maximum expiratory pressure; MIP: maximum inspiratory pressure.

**Table 5:** Descriptive statistics of cardiopulmonary exercise test measurements of male and female endurance athletes.

Characteristics	Female				Male			
	Min.	Max.	$\bar{X}$	SD	Min.	Max.	$\bar{X}$	SD
Max Speed (km/h)	13	16	14.91	0.79	13	18	16.47	1.03
MaxVO2 (mL/min/kg)	42.90	72.70	56.44	7.40	42.90	81.00	60.43	6.98
Max Time (min)	12:15	16:15	14:23	1:02	13:34	19:20	16:51	1:08
Max RQ	1.09	1.39	1.22	0.08	0.85	1.38	1.20	0.11
Rating of Perceived Exertion	4	10	5.70	2.77	3	10	5.60	2.3
VT-Time (min)	5:45	11:40	9:11	1:18	6:40	14:10	10:59	1:44
RC-Time (min)	8:55	14:00	11:47	1:16	7:40	16:45	13:52	1:45
VT-Speed (km/h)	9	13	11.22	0.85	10	14	12.40	1.17
RC- Speed (km/h)	11	14	12.96	0.82	10	16	14.36	1.24
VT-HR (puls)	102	195	172.57	18.28	123	194	166.96	14.93
RC-HR (puls)	101	206	182.43	20.62	124	211	178.26	14.69
Max-HR (puls)	184	205	199.35	5.82	164	206	194.70	8.94
Average Respiratory Frequency (1/min)	32.06	50.56	42.41	5.40	31.05	56.67	42.50	5.59

**Table 6:** Descriptive statistics of ergospirometric variables included in the regression analyses.

Characteristics	Female		Male	
	$\bar{X}$	SD	$\bar{X}$	SD
RCPCO2 (mL/dk)	4075.16	657.55	3509.31	773.89
RCPO2 (mL/dk)	3821.92	556.84	3413.52	721.65
VTCO2 (mL/dk)	3276.86	471.66	2797.47	635.29
VTO2 (mL/dk)	3418.63	522.59	3012.44	624.34
Maks Load PETO2 (mmHg)	102.91	2.55	102.91	4.37
Maks Load PETCO2 (mmHg)	36.56	3.3	36.12	4.23
VTidal (L)	2.51	0.39	2.14	0.48
Maks Load VE/VCO2	30.68	2.19	30.82	4.38
Maks Load VE/VO2	37.85	2.84	36.91	5.22
MaksVE (L/dk)	151.32	17.17	129.95	26.41

**Table 7:** Association between respiratory muscle strength and cardiopulmonary exercise variables in female and male endurance athletes.

Characteristics		Female				Male			
		*R*	*R* ^2^	Beta	*p*	*R*	*R*	Beta	*p*
Max Load VE	MEP	0.214	0.046	−0.105	0.769	0.327	0.107	0.234	0.155
	MIP			0.287	0.426			0.143	0.38
Max Load VE/VO2	MEP	0.1	0.01	−0.098	0.789	0.349	0.122	−0.241	0.139
	MIP			−0.003	0.994			−0.163	0.313
VTVCO2	MEP	0.281	0.079	−0.045	0.899	0.467	0.218	0.278	0.073
	MIP			0.315	0.375			0.267	0.084
Max Load VE/VCO2	MEP	0.468	0.219	0.518	0.030 *	0.255	0.065	−0.258	0.125
	MIP			−0.748	0.12			0.006	0.971
Max Load VTidal	MEP	0.183	0.034	−0.003	0.993	0.445	0.198	0.084	0.585
	MIP			0.186	0.606			0.399	0.012 *
Max Load PETCO2	MEP	0.535	0.286	0.864	0.010 *	0.433	0.187	0.449	0.006 *
	MIP			−0.665	0.042 *			−0.037	0.81
Max Load PETO2	MEP	0.318	0.101	−0.503	0.158	0.434	0.188	−0.387	0.016 *
	MIP			0.332	0.344			−0.085	0.585
MaxVO2	MEP	0.583	0.34	0.47	0.125	0.324	0.105	0.342	0.040 *
	MIP			0.136	0.647			0.043	0.79
RCP VCO2	MEP	0.198	0.039	−0.135	0.708	0.457	0.209	0.236	0.127
	MIP			0.285	0.43			0.296	0.050 *
RCP VO2	MEP	0.24	0.058	−0.237	0.506	0.495	0.245	0.314	0.040 *
	MIP			0.376	0.296			0.262	0.084
VT VO2	MEP	0.304	0.093	−0.362	0.306	0.467	0.25	0.28	0.065
	MIP			0.49	0.17			0.303	0.047 *

*: *p* < 0.05, VO2: Oxygen volume, VCO2: Carbon dioxide volume, VT: Respiratory threshold VE: Respiratory volume; RCP: Respiratory compensation point, PETCO2: End tidal carbon dioxide pressure, PETO2: End tidal oxygen pressure, VTidal: Tidal volume, Max Load: Maximum load, RCPVCO2: Carbon dioxide volume at Respiratory Compensation Point, RCPVO2: Oxygen volume at Respiratory Compensation Point, MaxVO2: Maximal oxygen consumption, Max Load VE/VO2: Ratio of O_2_ consumption volume at maximum load to respiratory volume, Max Load VE/VCO2: Ratio of CO_2_ production volume at maximum load to respiratory volume, VTVCO2: Volume of CO_2_ produced at respiratory threshold, VT VO2: Volume of O_2_ consumed at respiratory threshold. β = standardized regression coefficient. Adjusted *p*-values were calculated using the Benjamini–Hochberg False Discovery Rate (FDR) procedure.

## Data Availability

The original contributions presented in this study are included in the article. Further inquiries can be directed to the corresponding author.
